# The malonyl-CoA synthetase ACSF3 is required for metabolic editing downstream of biotin carboxylation

**DOI:** 10.1016/j.jbc.2026.113648

**Published:** 2026-10-14

**Authors:** Joseph Choi, Jasmine Encarnacion, Daria Kurganova, Susanna Scafidi, Michael J. Wolfgang

**Affiliations:** 1 Department of Physiology, Pharmacology & Therapeutics, Baltimore, Maryland USA; 2 Anesthesiology and Critical Care Medicine, The Johns Hopkins University School of Medicine, Baltimore, Maryland, USA

**Keywords:** mitochondrial metabolism, metabolic disease, proteomics, metabolomics, biotin, tricarboxylic acid cycle

## Abstract

Acsf3, a mitochondrial malonyl-CoA synthetase, is mutated in humans with inborn error Combined Malonic and Methylmalonic Aciduria. To clarify its role in mammalian metabolism, we generated Acsf3 knockout mice. We show that the loss of Acsf3 results in high concentrations of malonate and methylmalonate and exhibits expected defects in mitochondrial metabolism, but the KO mice are largely healthy, similar to the human inborn error. Acsf3 KO mice exhibit redundancy in malonyl-CoA synthetase activity, suggesting a genetic modifier of disease severity. Malonate and methylmalonate are metabolic poisons generated as a by-product of biotin-dependent carboxylation that require metabolic editing. Indeed, the liver-specific loss of the biotin ligase, Holocarboxylase Synthetase (hlcs), inhibits all biotin-dependent carboxylation and results in broad changes in liver mRNA, protein, posttranslational, and metabolic regulation. Loss of Hlcs suppresses the metabolic deficits in Acsf3KO liver due to the inhibition of malonate and methylmalonate generation. Mice with a double knockout of Acsf3 and Hlcs maintained mitochondrial lipoylation, demonstrating the highly redundant generation of mitochondrial malonyl-CoA. These data define the role of Acsf3 in metabolic editing of malonate and methylmalonate to maintain mitochondrial metabolism in the face of endogenously produced anti-metabolites.

Biotin is an essential water-soluble vitamin (B7) that is required for multiple carboxylases in central carbon metabolism that fix CO_2_ in the cytoplasm (Acetyl-CoA Carboxylase 1 and 2) or mitochondrial matrix (Pyruvate Carboxylase, Propionyl-CoA Carboxylase and Methylcrotonyl-CoA Carboxylase) (1). Acquired deficiencies or inborn errors in biotin accrual result in multiple carboxylase deficiency. Biotin is affixed to cytoplasmic and mitochondrial carboxylases in an ATP-dependent manner by a single enzyme, Holocarboxylase Synthetase (hlcs) 2, 3. Inborn errors in *hlcs* also result in multiple carboxylase deficiency and impact critical nodes in lipid, carbohydrate, and protein metabolism.

One of the important functions of biotin is the generation of malonyl-CoA, a central and well-described metabolite in fatty acid biochemistry (4). Malonyl-CoA is generated by the biotin-dependent carboxylation of acetyl-CoA by the highly regulated Acetyl-CoA Carboxylase (ACC). Eukaryotes encode two independent pathways for *de novo* fatty acid synthesis. The first is initiated by the biotin-dependent ACC localized canonically to the cytoplasm. The second is localized to the mitochondrial matrix, resembles prokaryotic fatty acid synthesis, generates medium-chain fatty acids and ultimately produces the critical cofactor α-lipoic acid 5, 6, 7, 8. Mitochondrial fatty acid synthesis is initiated, like the cytoplasmic pathway, by malonyl-CoA loading onto an acyl-carrier protein (ACP) (encoded by NDUFAB1, also a subunit of Complex I) by the malonyl-CoA ACP transacylase (MCAT) 9, 10. It then follows a similar set of reiterative biochemical reactions of elongation, reduction, and dehydration, ultimately generating octanoyl-ACP (C8:0-ACP). This is further processed to α-lipoic acid, which is loaded onto enzymes that require this essential cofactor, such as the glycine cleavage system, Pyruvate Dehydrogenase Complex, α-Ketoglutarate Dehydrogenase Complex, and key enzymes in amino acid catabolism (11). Beyond α-lipoic acid, it has been suggested that mitochondrial fatty acid synthesis generates other fatty acids that aid in electron transport assembly and function (12). While yeast express a mitochondrially targeted ACC, no such enzyme has been identified in mammals (13). Although malonyl-CoA is required for mitochondrial fatty acid synthesis, the source of mammalian malonyl-CoA remains undefined.

A *bona fide* source of mitochondrial malonyl-CoA is from the activation of malonate by ACSF3 (14). ACSF3 was originally described as an orphan member of the Acyl-CoA Synthetase (ACS) family of enzymes (15). The *Arabidopsis* ACSF3 ortholog, Acyl Activating Enzyme 13, was described as a eukaryotic malonyl-CoA synthetase essential for plant growth and viability, especially in the presence of exogenous malonate 16, 17. Like other acyl-CoA synthetases, ACSF3 ligates Coenzyme A to its co-substrates, malonate and methylmalonate, in an ATP-dependent manner. Mammalian ACSF3 localizes to the mitochondrial matrix to produce malonyl-CoA and methylmalonyl-CoA from malonate and methylmalonate within that organelle (18). Mitochondrial (methyl)malonate is an endogenous metabolite that is a classic competitive inhibitor of succinate dehydrogenase, a component of the tricarboxylic acid (TCA) cycle (19). As such, malonate is often used as a metabolic toxin to suppress oxidative metabolism 20, 21, 22. This suggests that a main function of Acsf3 is in metabolic editing, the detoxification of metabolites emanating from side reactions of other pathways. Mutations in human Acsf3 cause Combined Malonic and Methylmalonic Aciduria (CMAMMA) resulting in developmental delay, seizure disorders, hypoglycemia, and cardiomyopathy 23, 24. This phenotype is not fully penetrant in humans. Some individuals with null ACSF3 mutations present with the biochemical signature of CMAMMA but with a mild or benign clinical presentation 25, 26, 27. Nonetheless, the clinical phenotype of people with a genetic loss of ACSF3 is not consistent with a loss of lipoylation and the loss of ACSF3 in cell models does not result in lipoylation defects (28).

Here we have generated mouse models with a loss in Hlcs, Acsf3 or both to understand the role, requirement and interaction of biotin dependent carboxylation and mitochondrial malonyl-CoA synthesis. We demonstrate robust regulation and interaction at the mRNA, protein and metabolite level. A by-product of biotin carboxylation is the generation of metabolic poisons that must be resolved. These data define the role of Acsf3 in metabolic editing of malonate and methylmalonate to maintain mitochondrial metabolism.

## Results

### The acute liver-specific loss of *hlcs* is tolerated

HLCS ligates biotin onto the four mammalian carboxylases, Acetyl-CoA Carboxylase 1 and 2 (ACC) Pyruvate Carboxylase (PC), Propionyl-CoA Carboxylase (PCC), and Methylcrotonyl-CoA Carboxylase (MCC) in an ATP-dependent manner (Fig. 1*A*). To understand the impact of acutely limiting biotin dependent carboxylases specifically in the liver, we used an AAV vector driving iCre from the TBG promoter to delete *hlcs* specifically in the liver. We injected 5-week-old Hlcs^f/f^ mice with AAV8-TBG-eGFP (control) or AAV8-TBG-iCre and collected mice at 10 weeks in both the 4-h (fed) and 24-h (fasted) states. We chose fed and fasted states to understand the impact of HLCS on both anabolic and catabolic physiological states. The induced knockout of *hlcs* in the liver resulted in the loss of *hlcs* mRNA (Fig. 1*B*) and a striking absence of biotin on biotin-dependent carboxylases in the liver, as expected (Fig. 1*C*). This loss of biotin carboxylation did not, however, produce a discernable change in the abundance of oxidative phosphorylation complexes. (Fig. 1*C*). Accordingly, the gross morphology of the liver was also indistinguishable from controls under these conditions (Fig. 1*D*). Interestingly, the loss of carboxylases did not affect lipoylation of DLAT or DLST (Fig. 1*C*). These data indicate that unlike single-celled yeast, archaea, and bacteria, mammalian livers do not require cell-autonomous biotin carboxylation to generate lipoic acid *via* mitochondrial fatty acid synthesis.

**Figure 1:**
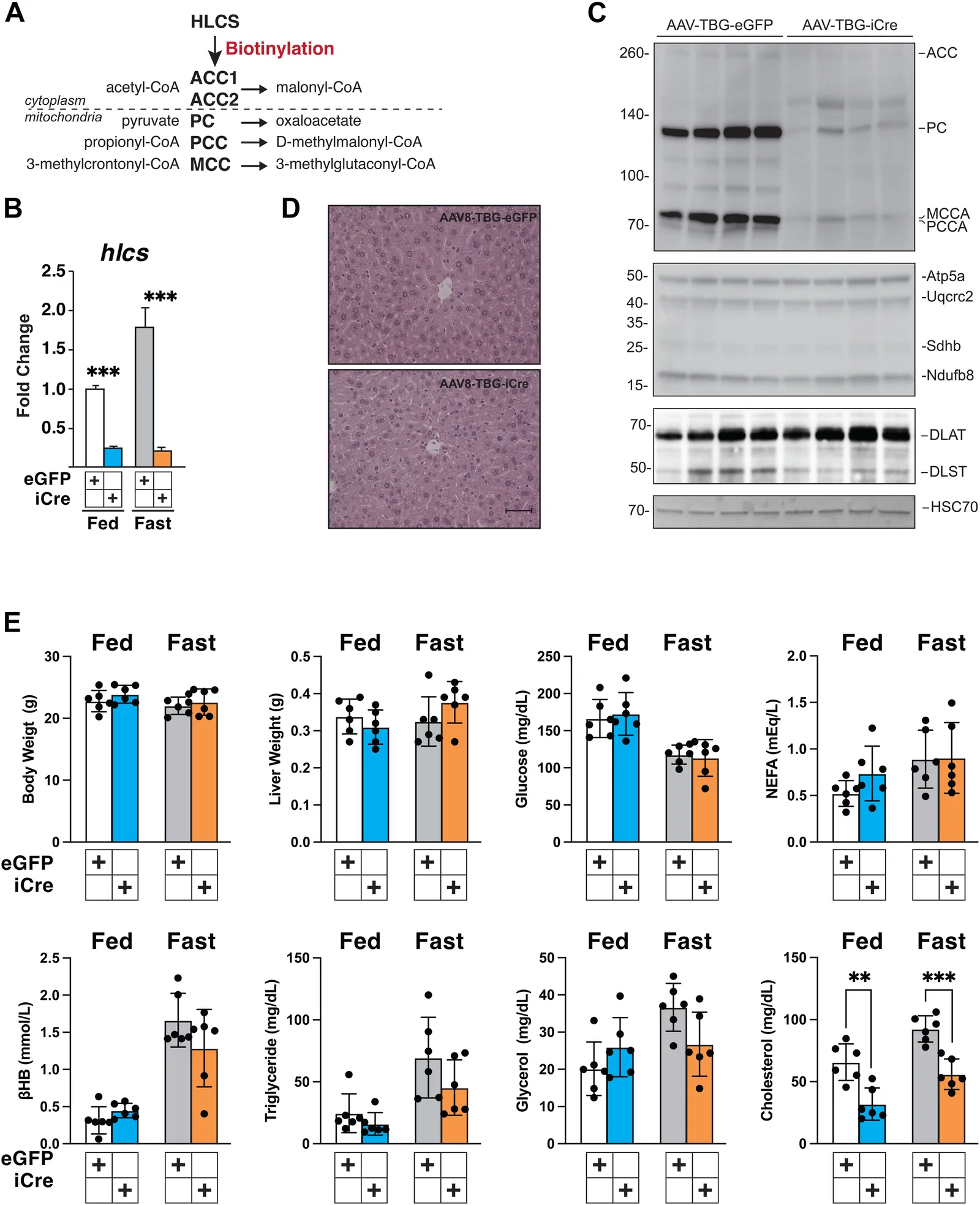
**Generation of mice with a liver-specific deficiency in biotin-dependent carboxylation.***A*, illustration of the enzymes biotinylated by HLCS in the cytoplasm (Acetyl-CoA Carboxylase 1 and 2) and in the mitochondria (Pyruvate Carboxylase, Propionyl-CoA Carboxylase, and Methylcrotonyl-CoA Carboxylase). *B*, RT-qPCR validation of the hlcs knockout induced by iCre compared to eGFP-injected controls. Liver hlcs mRNA from mice fasted for either 4 h (“Fed” state) or 24 h (“Fast” state) is shown. *C*, Western blot for liver biotinylated proteins (*upper*), oxidative phosphorylation complexes (*middle*), and lipoylated proteins (*lower*) in liver homogenate from eGFP and iCre-injected mice, with HSC70 as a control (*lowest*). *D*, H&E images of eGFP and iCre-injected mice. Scale bar represents 50 um (*E*) Body weights, liver weights, blood glucose measurements, and serum assays (non-esterified fatty acids, beta-hydroxybutyrate, triglycerides, glycerol, and cholesterol) for fed and fasted mice injected with either eGFP or iCre (n = 6/genotype).

The liver-specific loss of *hlcs* did not impact body weight, liver weight, blood glucose levels, ketone bodies, triglycerides, or glycerol levels in the fed or fasted state (Fig. 1*E*). The only difference observed in *hlcs* knockouts compared to controls is a small suppression of serum cholesterol independent of fasting state. Although biotin carboxylation is important for carbohydrate, lipid, and protein metabolism, and although the liver is a hub for regulating systemic metabolism in both the fed and fasted states, the loss of *hlcs* in the liver results in surprisingly mild systemic physiological deficits.

### Proteomics and transcriptomics of HLCS-deficient livers identify a dysregulated program for *de novo* lipogenesis

To understand the cellular impact of a loss in *hlcs* in the liver and gain insight into metabolic resilience in the absence of biotin-dependent carboxylation, we subjected the livers of 24 h fasted eGFP and iCre injected *hlcs* floxed mice to global TMT-proteomics. This analysis showed an upregulation of proteins related to *de novo* fatty acid synthesis including Acly, Fasn, Scd1 and G6pdx (Fig. 2*A*). The induction of Fasn protein was robust enough to be observed by Coomassie staining and was further validated by Western blot analysis (Fig. 2, *B* and *C*). The proteins induced by the loss of *hlcs* are canonically suppressed by fasting; therefore, to understand their regulation during times when the proteins are normally high, we compared mice in the 4 h and 24 h fasted states. Again, the loss of *hlcs* resulted in a reduction of biotin-dependent carboxylases that was exacerbated by extended fasting (Fig. 2*D*). While fasting suppresses DLAT and DLST lipoylation, a loss in *hlcs* produced no genotypic differences (Fig. 2*E*). Based on the proteomics data, we analyzed the abundance of Fasn, Acss2, Scd1 and Acly by Western blot in the fed and fasted states (Fig. 2*F*). As expected, genes in *de novo* fatty acid synthesis are elevated in the fed state and suppressed in the fasted state for both controls and knockouts; however, the induction of these proteins following *hlcs* deletion is particularly robust in the fed state. These data illustrate the altered regulation of *de novo* fatty acid synthesis with deficient hepatic biotin carboxylation.

**Figure 2:**
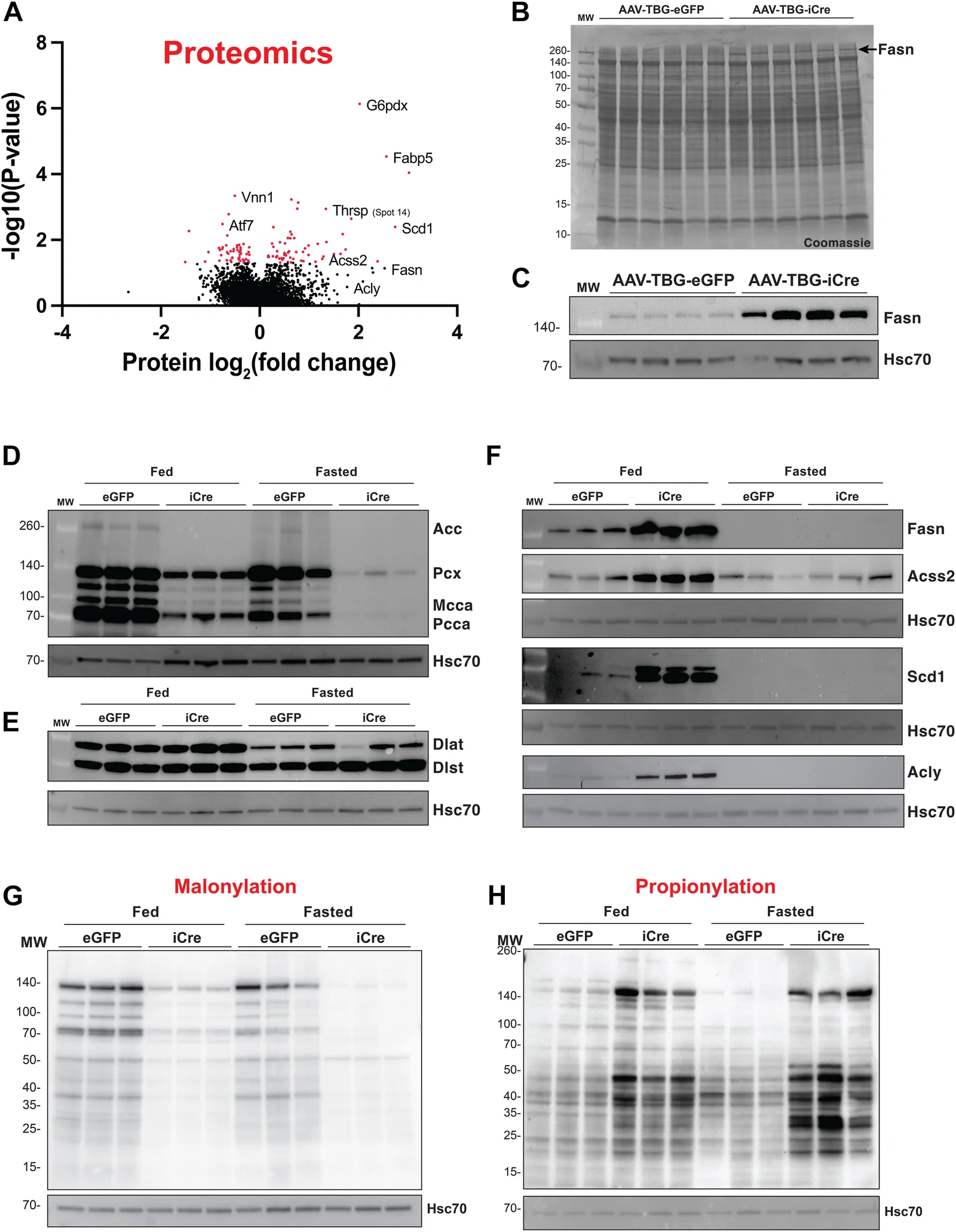
**Proteomics identified a dysregulated *de novo* lipogenesis program in hlcs-deficient liver.***A*, Volcano plot depicting significantly changed proteins between eGFP and iCre-injected groups in red (n = 5). *B*, coomassie stain for total liver protein, with the upregulation of Fatty Acid Synthase (Fasn) labeled. *C*, Western blot for Fasn in liver homogenate of eGFP and iCre-injected mice, using HSC70 as a loading control. *D–H*, Western blots for biotinylated proteins (*D*), lipoylated proteins (*E*), Fasn, Acyl-CoA synthetase short-chain family member 2 (Acss2), Stearoyl-CoA desaturase 1 (Scd1), ATP citrate lyase (Acly) (*F*), malonylated lysine (*G*), and propionylated lysine (*H*) in liver homogenates of fed and fasted eGFP and iCre-injected mice, using HSC70 as a loading control throughout.

The loss of Hlcs has a broad impact on liver metabolism; in particular, the major pathways for generating malonyl-CoA and for utilizing propionyl-CoA are both obstructed. We would therefore anticipate changes associated with lysine acylation in the absence of Hlcs. Indeed, the loss of Hlcs suppresses liver lysine malonylation, an effect which is further exasperated by fasting (Fig. 2*G*). Additionally, liver lysine propionylation is induced due to a block in propionyl-CoA utilization, as expected (Fig. 2*H*) (29). These data demonstrate broad changes in liver protein and posttranslational modifications following the loss of biotin carboxylation.

The deletion of *hlcs* in the liver leads to the loss of Acetyl-CoA Carboxylase activity, the rate-determining step in fatty acid synthesis. The differentially regulated proteins in the loss of *hlcs* closely resemble the impact of an ACC1/2 double knockout in the liver 30, 31. It has been suggested that malonyl-CoA-dependent fatty acid elongation generates poly-unsaturated fatty acids (PUFA) that, in turn, suppress the SREBP-dependent transcriptional regulation of fatty acid synthesis genes 30, 31. This seems to match our data very well. To examine the impact of *hlcs* deletion on transcriptional control in the liver, we performed RNA-seq analysis on livers from GFP- and iCre-injected mice collected in the fed state, when SREBP1 would be active. Indeed, we observed increases in genes known to be regulated by SREBP1, including Scd1, Fabp5, and Acss2 (Fig. 3*A*). We also observed a significant increase in the heptokine Gdf15, which is induced upon metabolic stress; this is also seen when the biotin-dependent pyruvate carboxylase is deleted (32).

**Figure 3:**
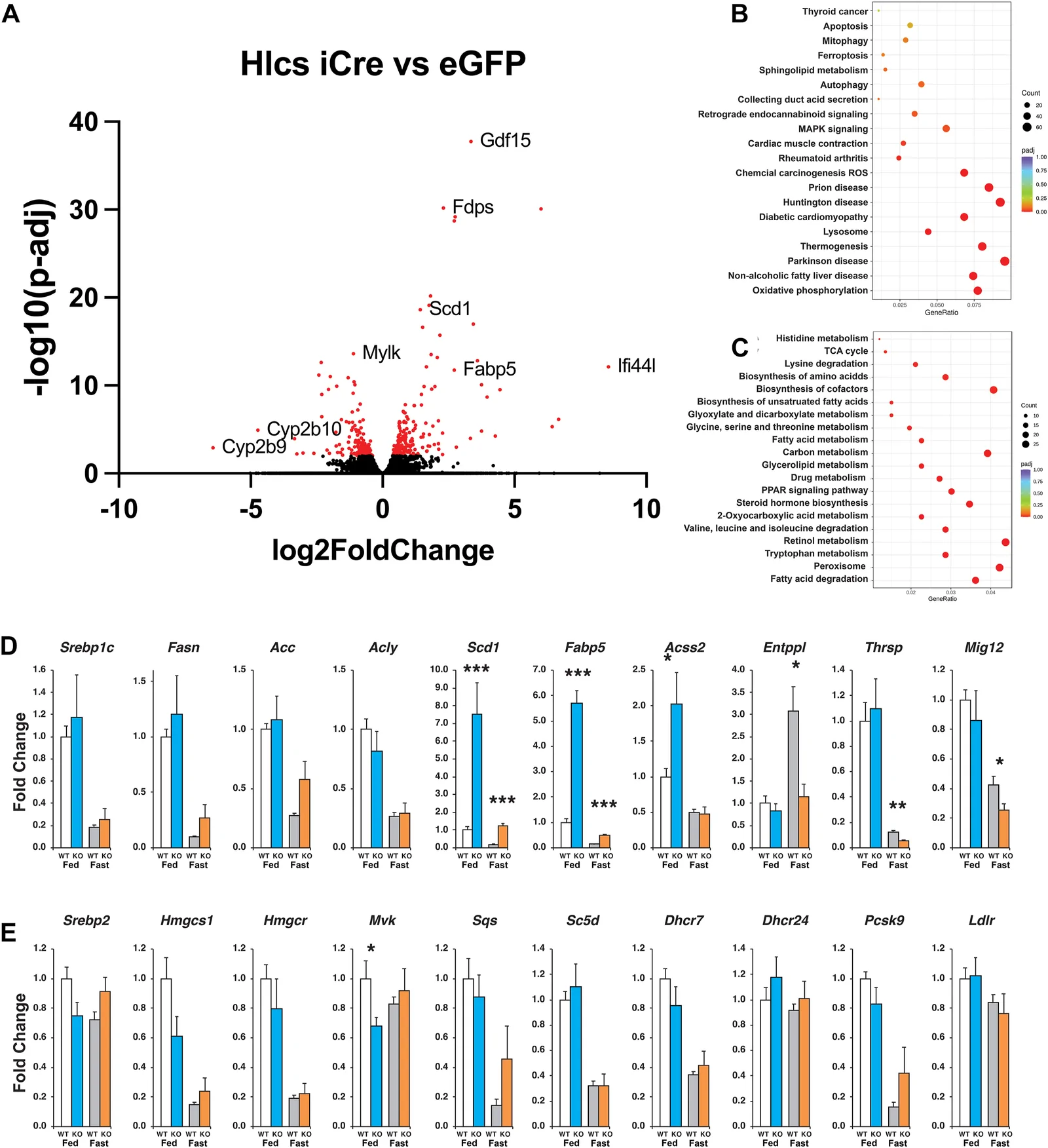
**Transcriptomics of HLCS deficient liver.***A*, RNAseq volcano plot depicting significantly changed genes between 4-h fasted eGFP and iCre-injected mice in *red* (n = 4). *B and C*, KEGG enrichment plots highlighting downregulated (*B*) and upregulated (*C*) gene pathways. *D*, gene expression (RT-qPCR) of Srebp1c, Fasn, Acc, Acly, Scd1, Fabp5, Acss2, Entppl, Thrsp, and Mig12 in livers of fed and fasted mice injected with either eGFP or iCre. *E*, gene expression of Srebp2, Hmgcs1, Hmgcr, Mvk, Sqs, Sc5d, Dhcr7, Dhcr24, Pcsk9, and Ldlr as measured by RT-qPCR (n = 6).

These data are consistent with a role of ACC-dependent regulation of SREBP.

The KEGG pathway analysis demonstrated a broad induction in fatty acid biosynthetic gene expression and a suppression in oxidative metabolism-related genes (Fig. 3, *B* and *C*). Surprisingly, this analysis did not reveal differences in *Fasn*, *Srebp*, or *Acly* mRNA expression, even though we observed robust differential regulation of their cognate proteins by proteomics and Western blot. Thus, we compared liver mRNA in the fed and fasted states from *hlcs*-floxed mice expressing eGFP or iCre by targeted qPCR.

Indeed, while *Scd1*, *Fabp5* and *Acss2* showed both Hlcs and metabolic state-specific regulation, *Srebp1*, *Fasn*, *Acc* and *Acly* demonstrated metabolic regulation but not Hlcs-dependent regulation (Fig. 3*D*). *Etnppl*, *Thrsp*, and *Mig12*, genes involved in other areas of fatty acid regulation, are downregulated in the livers of 24 h fasted Hlcs knockouts. The expression of cholesterol biosynthetic genes regulated by SREBP2 were not regulated, except for an increase in *Fdps* (Fig. 3, *A* and *F*). These data suggest that fatty acid biosynthetic enzymes are regulated by a combination of transcriptional and posttranscriptional mechanisms in the liver following the loss of biotinylated carboxylases.

### ACSF3 KO mice recapitulate combined malonic and methylmalonic aciduria

The biotin-dependent carboxylation of acetyl-CoA by ACC and propionyl-CoA by PCC generates malonyl-CoA and methylmalonyl-CoA, respectively. The free acids of malonyl-CoA and methylmalonyl-CoA are malonate and methylmalonate; these present a metabolic challenge, as both acids are TCA cycle poisons and competitive inhibitors of Succinate Dehydrogenase (SDH). Free malonate and methylmalonate can be activated to their CoA-esterified forms (and therefore detoxified) by Acsf3, a mitochondrially targeted acyl-CoA synthetase (14). Consequently, mutations in the *Acsf3* gene cause the human inborn error known as Combined Malonic and Methylmalonic Aciduria, or CMAMMA (24). This condition varies in severity and time of onset depending on the nature of the mutation, but the external factors determining how CMAMMA presents, from asymptomatic or lethal, are yet to be understood 23, 27.

To understand the physiologic ramifications of the inability to detoxify malonate and methylmalonate, we generated Acsf3 knockout mice. Using Crispr/Cas9, we introduced a stop codon to the initial tyrosine in the conserved AMP-binding domain, which is essential for acyl-CoA synthetases (Fig. 4*A*). This generated mice with an absence of Acsf3 mRNA (Fig. 4*B*). and Acsf3 protein (Fig. 4*C*). Accordingly, the mice exhibit an increase in serum and liver malonate and methylmalonate (Fig. 4, *D* and *E*). Otherwise, they exhibit normal characteristics with wild-type levels of NEFA and ketone bodies in both 4 h and 24 h fasting states (Fig. 4*F*). Subtle changes in serum triglyceride and cholesterol are present, with a significant increase in triglyceride and decrease in cholesterol found in the fed state only (Fig. 4*F*). Given the high circulating and tissue levels of the putative metabolic poisons, Acsf3 knockout mice remain surprisingly normal, consistent with the human phenotype.

**Figure 4:**
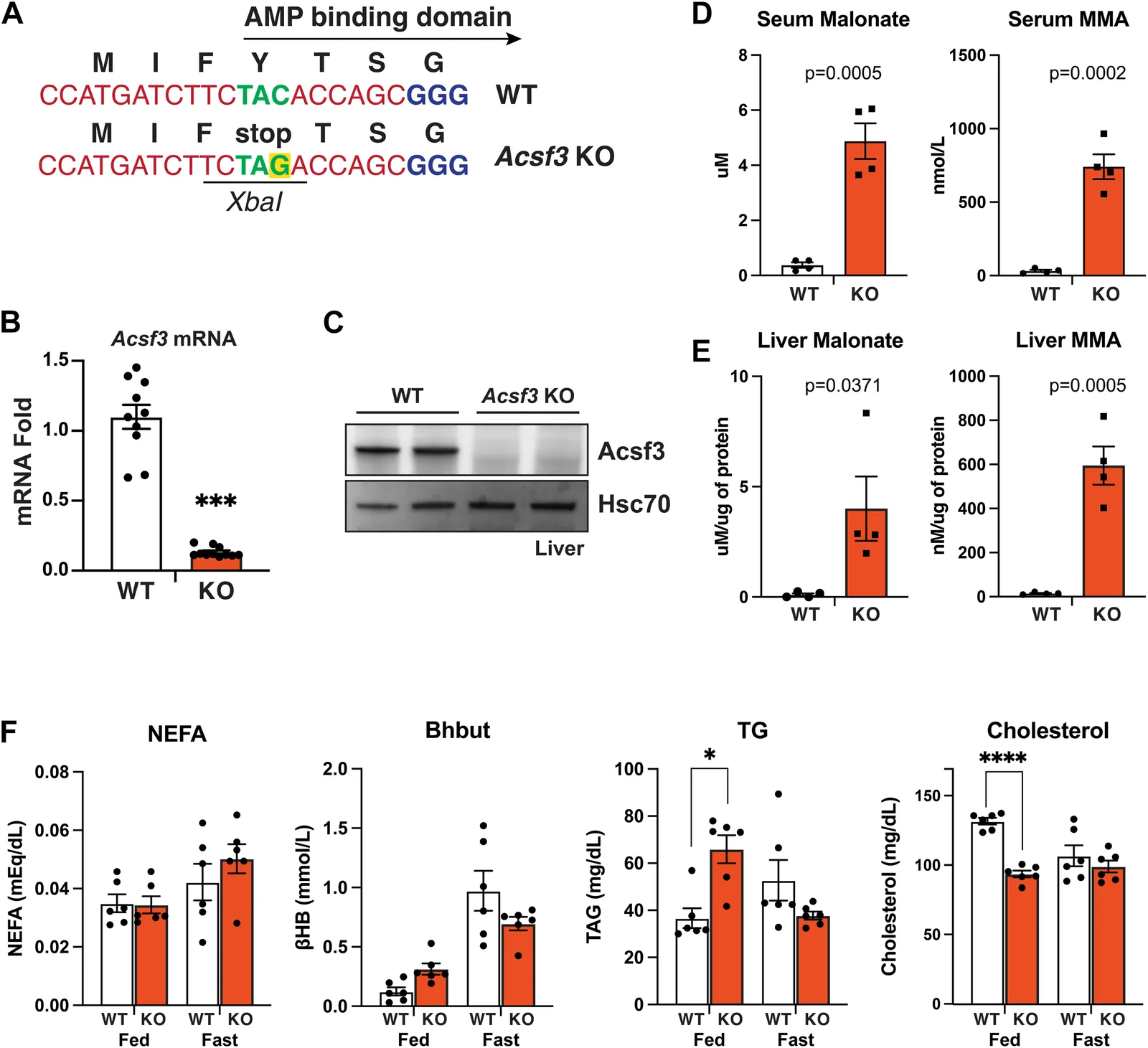
**ACSF3 knockout in mice recapitulates combined malonic and methylmalonic aciduria.***A*, illustrated generation of the ACSF3 mutation resulting in the loss of a critical catalytic domain (ACSF3 knockout). *B*, RT-qPCR measurement of ACSF3 mRNA in the livers of control (WT) and ACSF3 knockout (KO) mice, validating the ACSF3 knockout. *C*, Western blot indicating the loss of Acsf3 protein in liver homogenates of KO mice compared to WT. HSC70 is used as a loading control. *D and E*, malonate and methylmalonic acid (MMA) in the serum (*D*) and livers (*E*) of WT and KO mice. Significance determined by Student’s *t* test. *F*, serum lipid assays for non-esterified fatty acids (NEFA), beta-hydroxybutyrate (Bhbut), triglyceride (TG), and cholesterol in control (*white*) and ACSF3 knockout (*orange*) mice in the fed and fasted states (n = 6). Statistical significance was determined by Two-way ANOVA with Tukey’s Multiple Comparisons test, and statistical similarity is represented by letter.

### ACSF3 independent malonate and methylmalonate metabolism

To further characterize the metabolic effect of Acsf3 deletion, we profiled liver acylcarnitines in Acsf3 knockouts vs WT (Fig. 5*A*) as well as HLCS iCre livers vs eGFP (Fig. 5*B*). The largest changes in Acsf3KO liver acylcarnitines were stark increases in methylmalonyl-carnitine, and to a lesser extent, malonyl-carnitine. In contrast, these metabolites were suppressed in Hlcs iCre livers compared to their controls. The Acsf3 result was surprising, given that the CoA derivatized form is required to generate the carnitine esters. This suggests the presence of an alternative pathway to activate malonate and methylmalonate when their concentrations increase dramatically.

**Figure 5:**
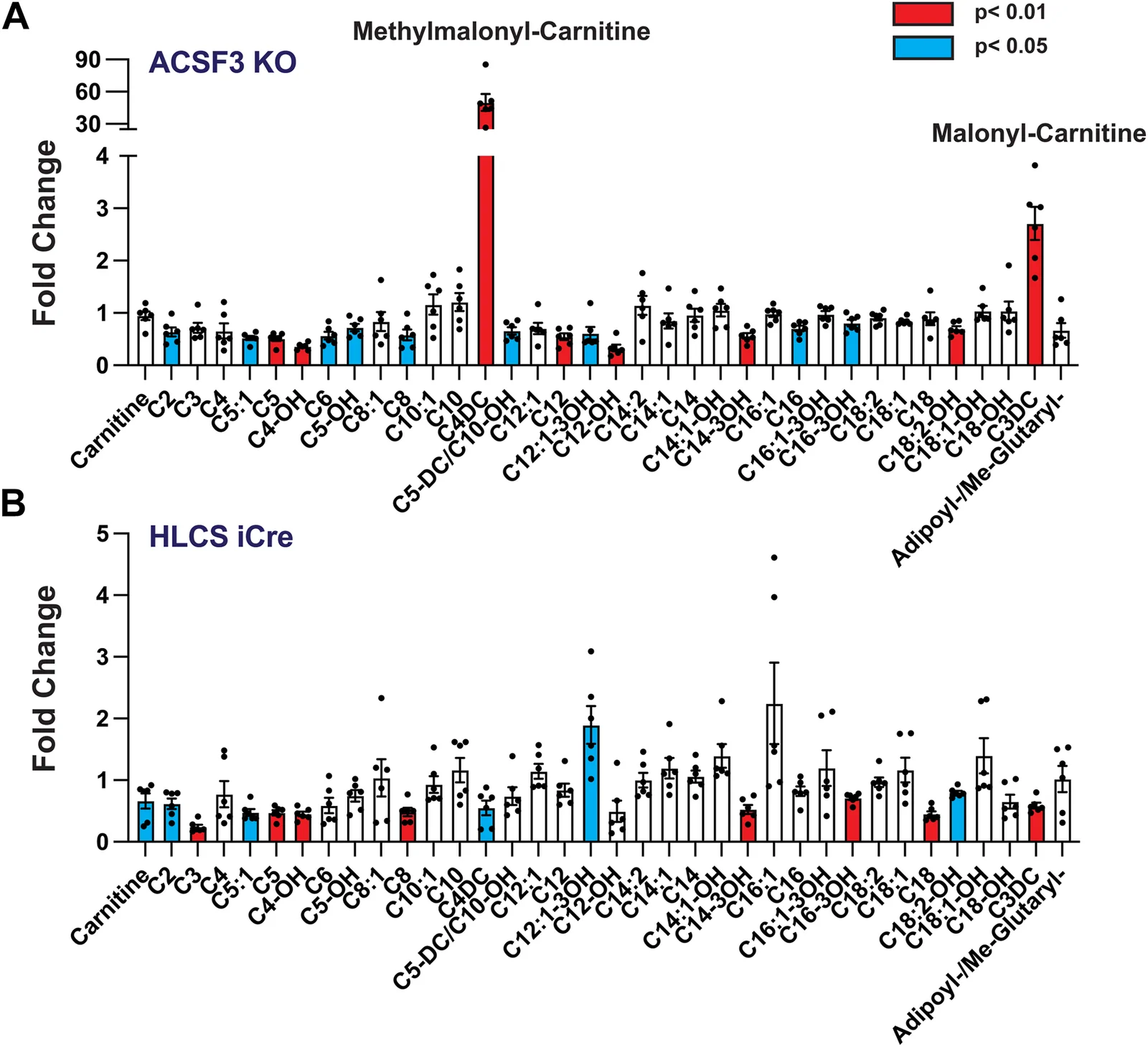
**ACSF3 knockout mice retain limited malonyl-CoA synthetase activity.***A and B*, fold change of liver acylcarnitines measured from ACSF3 knockout mice (*A*) and HLCS iCre-injected mice (*B*) compared to controls (n = 6). Significance determined by Student’s *t* test; ⁎⁎⁎⁎*p* < 0.0001.

### The loss of Hlcs suppresses Acsf3-mediated mitochondrial toxicity

Hlcs-mediated biotin dependent carboxylation and Acsf3 converge at the generation of malonyl-CoA and methylmalonyl-CoA. To rigorously probe this interface, we generated liver-specific double knockouts of both Hlcs and Acsf3 by crossing Hlcs^f/f^ and Acsf3^−/−^ mice and injecting these mice with AAV8-TBG-icre as above. This results in a whole-body deletion of Acsf3 and a liver specific deficit in Hlcs. Analysis of these genes in the livers of double knockouts confirmed mRNA levels analogous to Hlcs and Acsf3 single knockouts (Fig. 6*A*). A Western blot illustrates a decrease in biotin-dependent carboxylases, and an appropriate loss of Acsf3 protein in both the single and double knockouts (Fig. 6*B*). However, few changes were observed in circulating ketones and triglycerides, with a slight decrease in NEFA for the double knockouts compared to only the Hlcs single knockouts (Fig. 6*C*). Again, we see a suppression in serum cholesterol. These mice exhibit a loss in all the canonical pathways that generate malonyl-CoA, ACC1, ACC2 and ACSF3 in the liver.

**Figure 6:**
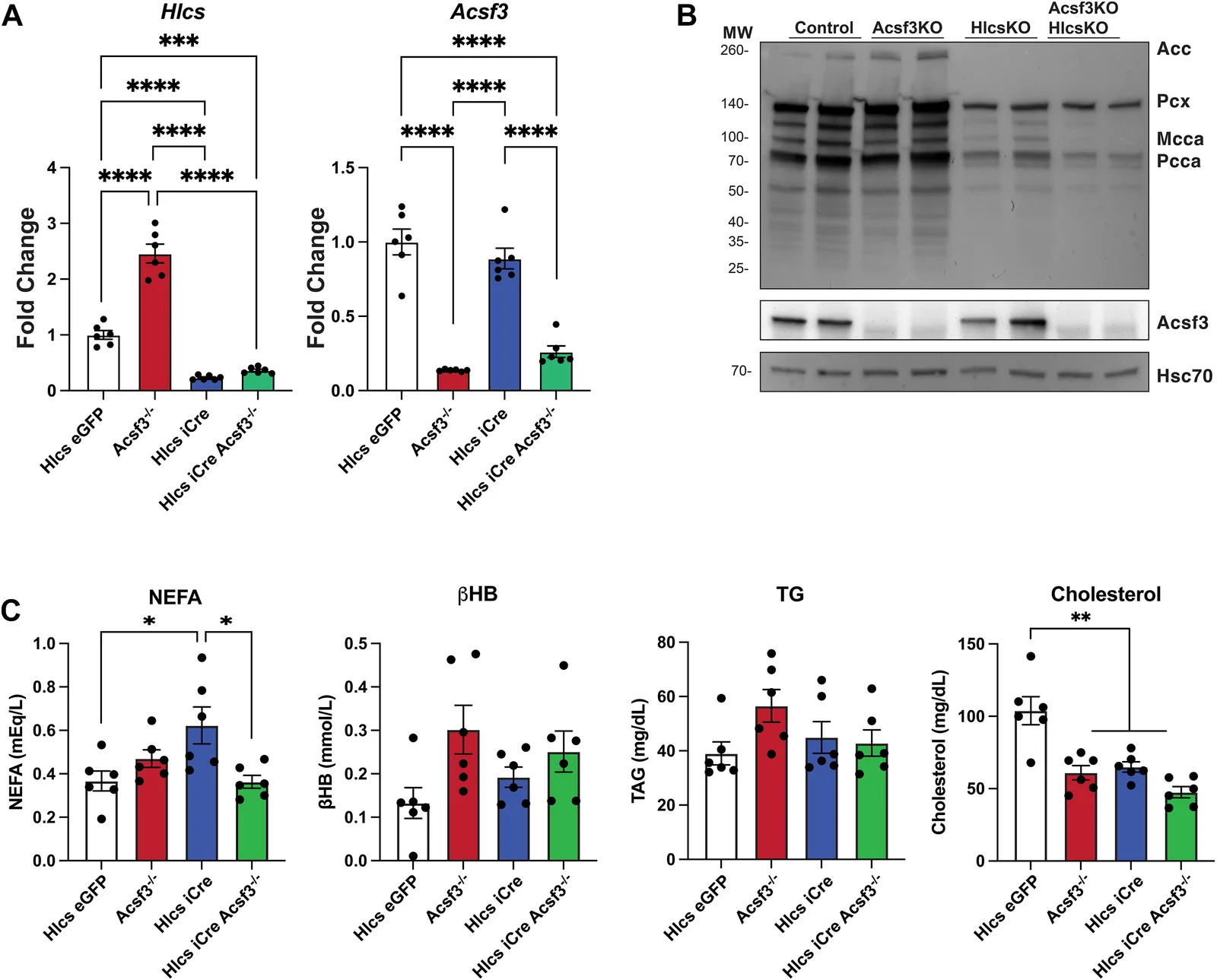
**Generation of liver-specific HLCS, ACSF3 double knockout mice.***A*, RT-qPCR fold-change of *HLCS* and *ACSF3* mRNA in the livers of ACSF3^−/−^ knockouts, HLCS iCre knockouts, and HLCS iCre/ACSF3^−/−^ double knockouts compared to controls (HLCS eGFP). Statistical significance determined by one-way ANOVA with Tukey’s multiple comparison test. Statistical similarity shown by letter. *B*, Western blot for liver proteins in control, ACSF3^−/−^, HLCS iCre, and HLCS iCre/ACSF3^−/−^ mice. Primary antibodies against biotin (*upper*), ACSF3 (*middle*) and HSC70 (*lower*) were used. *C*, serum lipid assays for non-esterified fatty acids (NEFA), beta-hydroxybutyrate (ßHB), triglyceride (TG), and cholesterol in control, ACSF3^−/−^, HLCS iCre, and HLCS iCre/ACSF3^−/−^ mice (n = 6).

We showed that the loss of Hlcs in the liver resulted in a large increase in the proteins of *de novo* fatty acid synthesis. Here we probed these genes in the four genotypes. Western blots demonstrate the levels of Acss2, Fasn, Acly, and Scd1 proteins found in the livers of Hlcs/Acsf3 double knockout mice compared to controls and single knockouts. Acss2 and Fasn levels in Hlcs/Acsf3 knockout livers are very similar to Hlcs single knockouts (Fig. 7*A*). The elevated Acly found in Hlcs single knockouts is reduced, while Scd1 is increased for double knockouts (Fig. 7*A*). Western blot for malonylation shows a similar phenotype for Hlcs single and Hlcs/Acsf3 double knockouts, compared to Acsf3 single knockouts, which appear analogous to controls (Fig. 7*B*). There was no change in the lipoylation of DLAT or DLST in any of the knockouts (Fig. 7*B*). These data show that ACC drives cellular malonylation but loss of all the known pathways for the generation of malonyl-CoA does not affect mitochondrial lipoylation.

**Figure 7:**
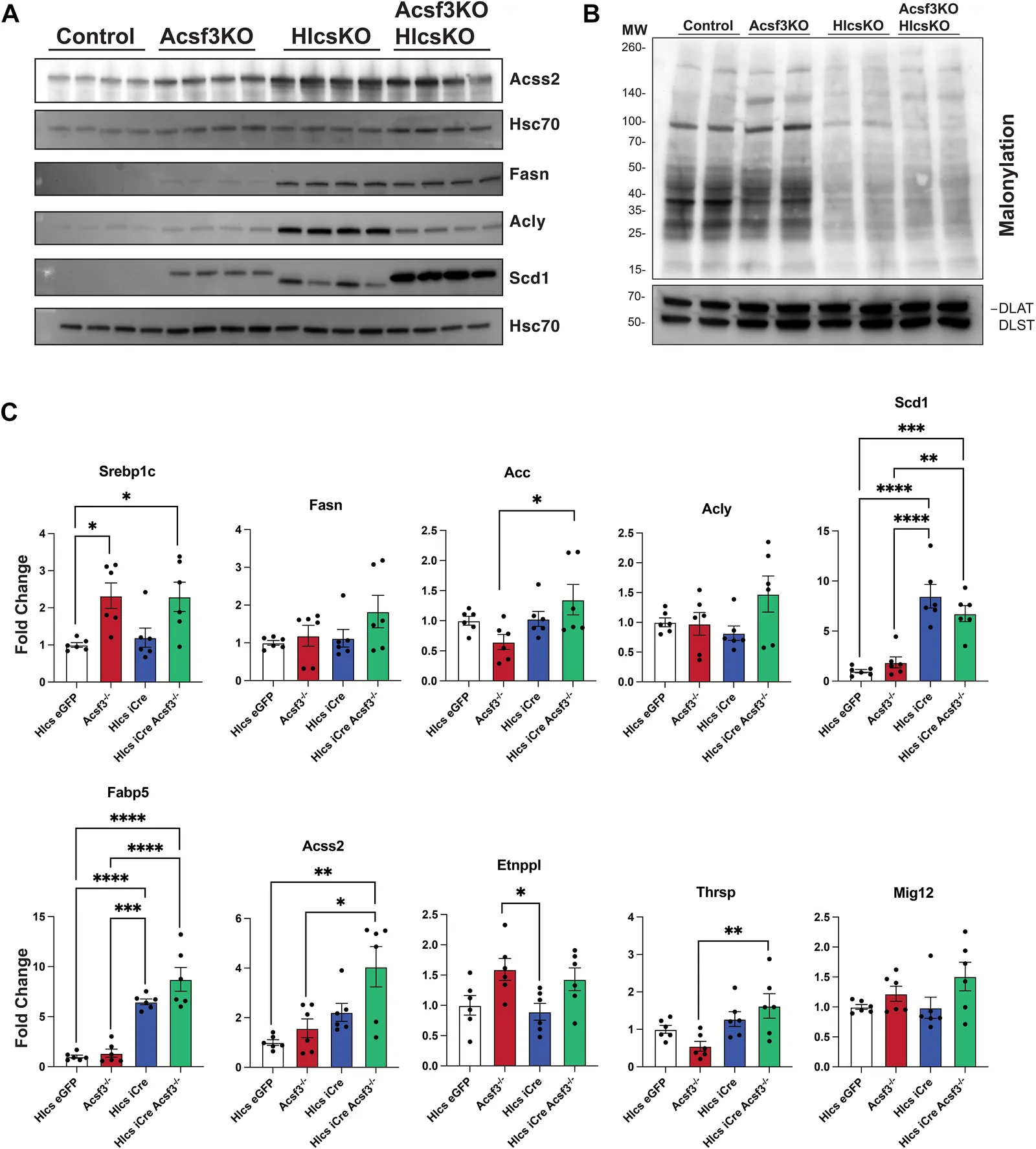
**Biotin carboxylation regulates fatty acid synthesis and lysine malonylation.***A*, Western blots for Acss2, Fasn, Acly, and Scd1 across control, ACSF3^−/−^, HLCS iCre, and HLCS iCre/ACSF3^−/−^ mouse livers (n = 4). HSC70 is used as a loading control. *B*, Malonylated lysine and lipoylation western blots for control, ACSF3^−/−^, HLCS iCre, and HLCS iCre/ACSF3^−/−^ mouse livers (n = 2). The locations of molecular weight markers (MW) are labeled. *C*, RT-qPCR gene expression measurements of Srebp1c, Fasn, Acc, Acly, Scd1, Fabp5, Acss2, Entppl, Thrsp, and Mig12 mRNA in livers of control (HLCS eGFP), ACSF3^−/−^, HLCS iCre, and HLCS iCre/ACSF3^−/−^ mice (n = 6). Statistical similarity (shown by letter) determined by one-way ANOVA with Tukey’s multiple comparison test.

Profiling the mRNA of liver proteins reveals a similar pattern to that found in Hlcs deficiency (Figs. 2 and 3), wherein mRNA measurements are not directly analogous to the observed protein levels. While *Acc* and *Acss2* mRNA suggest a more intense phenotype in DKOs compared to all other groups, *Fasn* and *Acly* fail to show any significant mRNA changes even in the face of large protein changes (Fig. 7*C*). Interestingly, although Scd1 protein is strongly increased in double knockouts compared to either single knockout group, the mRNA for Scd1 is not significantly changed from Hlcs single knockouts (Fig. 7*C*). These data further suggest a post-translational regulation of proteins involved in fatty acid metabolism in the absence of Hlcs, Acsf3, or both.

To gain a clearer understanding of the biochemical consequences and interactions in these two models, we examined the metabolomes Acsf3 and HLCS single- and double-knockout livers compared to controls (Fig. 8, *A*–*C*). In contrast to protein and mRNA data, metabolite analysis attributed the most pronounced phenotype to Acsf3 single knockouts. Principal component analysis demonstrates this. Acsf3 deviates from WT the most, and the double HLCS/ACSF3 double KO are more closely aligned with HLCS single KO and WT metabolomes (Fig. 8*D*). The loss of ACSF3 resembles a mitochondrial insufficiency. ACSF3KO liver exhibits large increases in glycolytic intermediates FBP, GAP, DHAP, and a suppression in citrate and alpha-ketoglutarate (Fig. 8, *E* and *F*). Interestingly, changes in glycolytic intermediates revert to wild-type levels with the combined deletion of Hlcs, when the substrates for ACSF3 are suppressed (Fig. 8*E*). The metabolomics data are consistent with the known biochemical functions of biotin carboxylation and malonyl-CoA synthetase, Acsf3 (Fig. 8*G*). That is, the role of Acsf3 is to detoxify TCA cycle poisons (metabolic editing) emanating from the products of biotin carboxylation.

**Figure 8:**
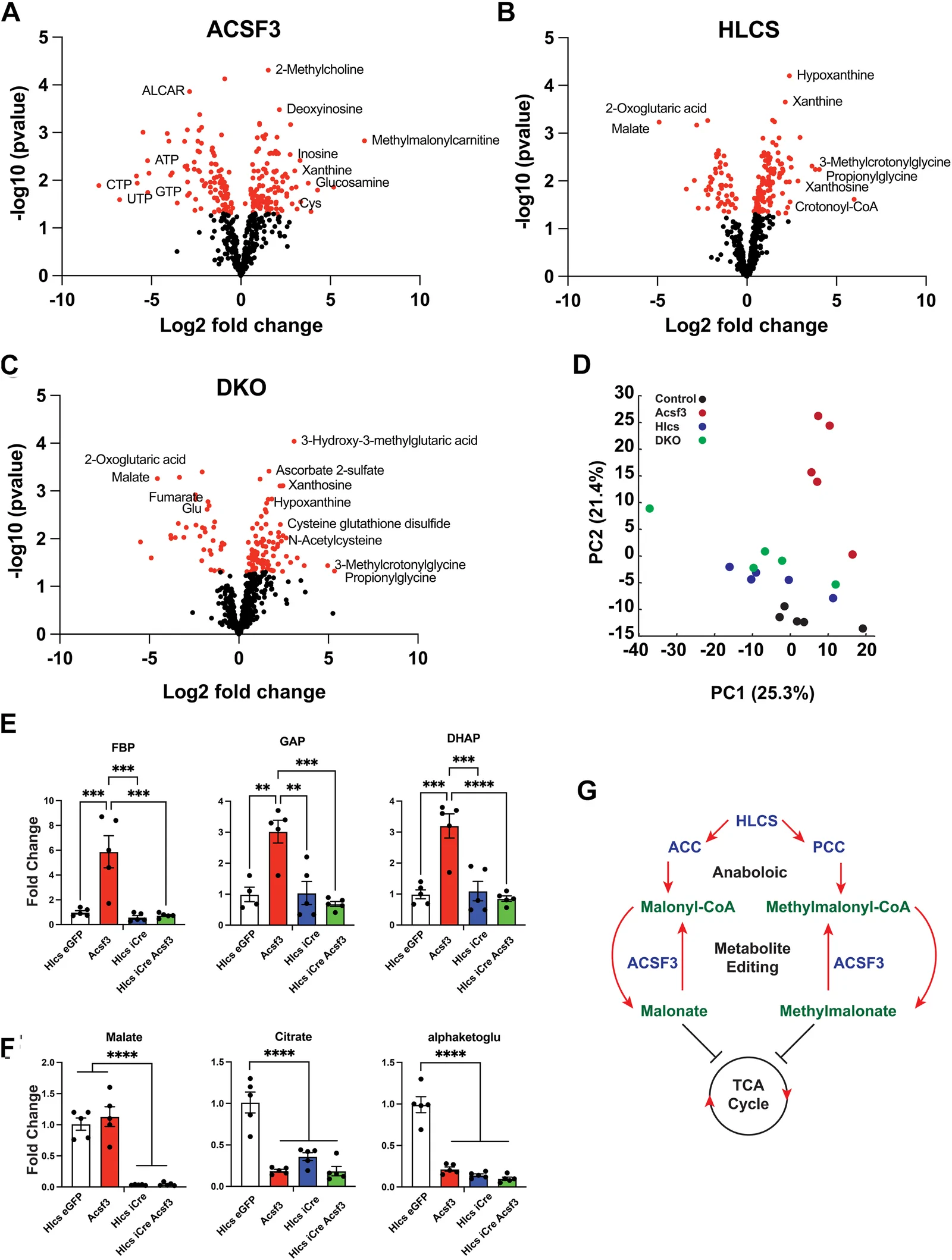
**The ACSF3 knockout liver metabolome resembles a mitochondrial insufficiency.***A-C*, Volcano plots highlighting significantly changed metabolites (*red*) in ACSF3^−/−^ (*A*), HLCS iCre (*B*), and HLCS iCre/ACSF3^−/−^ (DKO) mice (*C*) compared to controls (n = 5). *D*, PCA plot showing the deviations in metabolic profile across models. *E and F*, Bar graphs showing metabolite levels of FBP, GAP, and DHAP (*E*) or malate, citrate, and alpha-ketoglutarate (α-KG) (*F*) as the fold-change from control values in ACSF3^−/−^, HLCS iCre, and HLCS iCre/ACSF3^−/−^ mouse livers. Statistical similarity (shown by letter) determined by one-way ANOVA with Tukey’s multiple comparison test. *G*, illustration of the relationship between HLCS and ACSF3 activity in the metabolism of malonate and methylmalonate.

## Discussion

ACSF3 is a member of the acyl-CoA synthetase family encoded by 26/25 human/mouse genes that esterify fatty acids to CoA in an ATP-dependent manner (15). ACSF3 was deorphanized in *Arabidopsis* as a malonyl-CoA synthetase (16). The plants failed to thrive and accumulated malonic acid. Soon after, it was described as a gene mutated in humans with Combined Malonic and Methylmalonic aciduria, resulting in developmental delay, seizure disorders, hypoglycemia, and cardiomyopathy 23, 24. These patients presented with serious pathology. The metabolic logic is clear, as malonate and methylmalonate are mitochondrial inhibitors as free acids 20, 21, 22. Based on these studies, population-based screening was performed, and it was found that the phenotype is not fully penetrant in humans; some individuals with null ACSF3 mutations present with a mild or benign clinical presentation even though they exhibit elevated malonate and methylmalonate (27). This dichotomy between serious and mild disease suggests environmental and/or genetic modifiers of the disease.

Similar to humans with CMAMMA, the loss of *acsf3* in mice is not lethal 33, 34. However, *in vitro* studies suggest that its loss inhibits mitochondrial function as expected 28, 33. The liver metabolomics presented here also suggest that increased malonate and methylmalonate inhibit mitochondrial metabolism as expected, with suppressed TCA cycle intermediates and increased glycolytic intermediates. This rather dramatic evidence of mitochondrial disease does not lead to expected phenotypes in the mice. The rise in malonyl-carnitine and methylmalonyl-carnitine suggests there is redundancy within the short chain acyl-CoA synthetases, and this may be one genetic modifier to explain the phenotypic dichotomy in humans with CMAMMA. Nonetheless, the maintenance of systemic metabolic homeostasis in the face of mitochondrial toxins is remarkable.

Mammals encode two modes of fatty acid synthesis, one cytoplasmic and one mitochondrial. Both are critical for survival. Given its mitochondrial localization, it was shown that Acsf3 supported mitochondrial fatty acid synthesis (18). However, the human and mouse phenotypes do not resemble defects in mitochondrial fatty acid synthesis, and knockout cells do not show a defect in lipoylation, a product of mitochondrial fatty acid synthesis (28). Therefore, although Acsf3 can contribute to mitochondrial fatty acid synthesis it is not required. Mammalian cytoplasmic *de novo* fatty acid synthesis uses malonyl-CoA as the chain elongation unit derived from acetyl-CoA carboxylase. Yeast generates mitochondrial malonyl-CoA by a mitochondrial matrix-localized acetyl-CoA carboxylase (13). It has been suggested that mammals also express a variant of ACC1 that traffics to the mitochondrial matrix (35). However, the liver-specific double knockout of ACC1 and ACC2 eliminates fatty acid synthesis and fatty acid elongation but not mitochondrial fatty acid synthesis 30, 31. Therefore, unlike yeast, acetyl-CoA carboxylase is not required for mitochondrial fatty acid synthesis, at least not in a cell-autonomous manner.

Other organisms such as Archaea or the obligate intracellular bacteria *rickettsia* express a dual function carboxylase that can carboxylate acetyl-CoA or propionyl-CoA. Mammalian propionyl-CoA carboxylase can generate malonyl-CoA, albeit at a low efficiency (1). In order to eliminate the possibility that carboxylation in any form was required for lipoic acid synthesis, we targeted Holocarboxylase Synthetase for knockout specifically from the liver. We found that biotin carboxylation, which is necessary for the activity of ACC, PC, PCCA, and MCCA, is not required for mitochondrial fatty acid synthesis in a cell-autonomous manner. To investigate the interactions surrounding biotin carboxylation, we deleted both Acsf3 and Hlcs in the liver and again demonstrated that these 5 enzymes were not required for mitochondrial fatty acid synthesis. Evidently, the generation of mitochondrial malonyl-CoA is highly redundant for this essential pathway.

The loss of Hlcs suppresses the generation of malonyl-CoA and methylmalonyl-CoA, therefore reducing the requirement for Acsf3. Hlcs loss suppresses much of the mitochondrial disease signature, such as the rise in glycolytic intermediates in Acsf3 KO liver, demonstrating the requirement of Acsf3 in metabolic editing. The loss of Hlcs in the liver largely resembles the impact of ACC1/2 double knockout, including the induction of proteins in *de novo* fatty acid synthesis. There exists a disparity between the mRNA and protein levels of these enzymes. This is particularly striking for fatty acid synthase, which is highly elevated at the protein but not mRNA level, pointing to posttranslational regulation. Indeed, the loss of Hlcs induces opposite changes in lysine malonylation and propionylation. Interestingly, Acly (a cytoplasmic enzyme) is induced in Hlcs KO liver, but this is somewhat reversed in Hlcs/Acsf3 double KO liver while the mRNA is unchanged.

Here, we have generated *in vivo* models to understand the role of Acsf3 in organismal metabolic homeostasis. Like the human inborn error in Acsf3, the mouse knockout experiences a robust metabolic phenotype but can maintain physiologic homeostasis. The loss of biotin carboxylation by knocking out the biotin ligase, hlcs, suppresses the metabolic phenotype, clearly placing Acsf3 in metabolic editing. Neither Acsf3 or Hlcs individually or in combination were required for mitochondrial fatty acid synthesis pointing to the high-level redundancy for this ancient pathway.

## Experimental procedures

### Mouse models

Mice with a floxed *hlcs* gene were obtained from Jackson labs (Jax #035494) and crossed to R26-CreERT2 line 36, 37. Acsf3 KO mice were generated using Crispr/Cas9 CCATGATCTTCTACACCAGC to introduce a stop codon to the initial tyrosine in the conserved AMP binding domain, which is essential for acyl-CoA synthetases. The gRNA target was CCATGATCTTCTACACCAGC and the sequence for the ssDNA repair was GTTGAAGAGAGTGGGTGGCGTGACCGGGGTGCCATGATCTTCTAGACCAGCGGGACCACAGGGAGGCCCAAGGGTGCACTGAGCAC that introduced a stop codon and an XbaI site to facilitate genotyping. To induce full-body knockouts in ER^T2^ mice, we injected 5-week-old *hlcs* mice with tamoxifen (T5648-1G, Sigma-Aldrich) in a corn oil vehicle with 2% ethanol to improve solubility. Mice were given five intraperitoneal injections at 100 mg/kg over the course of 7 days. To induce liver-specific hlcs knockout in 5-week-old mice, we retro-orbitally injected $2\times 10^{11}$ genome copies of an adeno-associated virus vector (AAV8) containing either TBG-eGFP (VB1743, Vector Biolabs) or TBG-iCre (VB1724, Vector Biolabs) driven by the TBG promoter (29).

Mice were housed in ventilated racks with either a 12 hr-light/12 hr-dark cycle or 14 hr-light/10 hr-dark cycle and fed a standard chow diet (5V75, Labdiets). Before sacrifice, mice were fasted for either 4 h or 24 h with water provided *ad libitum*. All procedures were performed in accordance with the NIH’s Guide for the Care and Use of Laboratory Animals and under the approval of the Johns Hopkins School of Medicine Animal Care and Use Committee.

### Western blot

For liver protein western blotting, proteins were extracted using RIPA lysis buffer with cOmplete protease inhibitor (#11836170001, Roche) and PHOSSTOP phosphatase inhibitor (#4906847001, Roche), and tissues were fully lysed through bead homogenization. Concentrations of liver protein homogenates were assessed following Pierce BCA Protein Assay (#23227, Thermo Fisher). Samples were adjusted to 50 ug/20 ul and prepared using 4x Laemmli Sample Buffer (Bio-Rad) according to the manufacturer's protocol. 20 ul per well was loaded onto 4 to 15% Criterion TGX Precast Midi Protein Gels (Bio-Rad) and run at 200 V for approximately 40 m.

Protein was transferred from SDS-PAGE gels to PVDF membranes using the TurboBlot Transfer system (Bio-Rad). Ponceau S staining (#59803, Cell Signaling) for 10 min was used to verify consistent protein transfer. Following ponceau stain, membranes were washed thrice in Tris-buffered saline with Tween-20 (TBST) for 5 min each, and blocked with 3% BSA in TBST for 1 h at RT. After blocking and washing 3 × 5 min in TBST, blots were incubated with primary antibodies in 3% BSA overnight at 4 °C; Propionyllysine (Kpr) (PTM-201, PTM Biolabs), Biotin (Sigma, A4541), Mitoprofile (Abcam ab110413), FASN (BD Biosciences 610963), SCD1 (Cell signaling 2794), ACLY (Cell signaling 4332), malonyl-lysine (Cell Signaling 14942), α-lipoic acid (Calbiochem #437695), ACSF3 (ThermoFisher PA5-25803) and HSC70 (sc-7298, Santa Cruz). Blots were incubated with secondary antibodies in 5% milk for 2 h at RT: (Anti-Rabbit HRP, #7074S, Cell Signaling), HSC70 (Anti-Mouse Cy3, M30010, Thermo Fisher). Amersham ECL Select (Cytiva) was used on HRP-linked antibodies for imaging on an Alpha Innotech MultiImageIII instrument.

### Reverse transcription-quantitative PCR

Tissues were homogenized in Trizol reagent (11596018, Invitrogen) and RNA was isolated using the RNeasy Mini Kit (QIAGEN). Using the High Capacity cDNA Reverse Transcription Kit (Applied Biosystems), we reverse transcribed 1 to 2 μg of total RNA. RNA quality was assessed by Nanodrop. RT-qPCR was performed using SsoAdvanced Universal SYBR Green Supermix (RK21203, ABclonal Technology) following manufacturer recommendations. *Cyclophilin A* and *18s* were used to normalize Cq values. RT-qPCR data analysis was performed in Excel and GraphPad PRISM.

### Metabolic assays

Blood was collected in 1.5 ml Eppendorf tubes and placed on ice immediately following sacrifice of mice. Serum was isolated by centrifugation of blood for 10 min at 12,000×*g*, 4 °C. BHB was measured using the Stanbio B-hydroxybutyrate Liquicolor kit (2440-058). Non-esterified fatty acids in serum were assessed with the Wako HR series NEFA-HR(2) kit and associated standard solution (Fujifilm). Serum cholesterol was measured using the Fujifilm Cholesterol E kit (999-02601). Total triglyceride was measured using the Infinity Triglycerides Liquid Stable Reagent (TR22421). Data were collected using an Agilent Biotek Gen5 microplate reader and Imagine software (ver. 3.13) and processed using GraphPad PRISM.

### RNA sequencing

Total RNA was extracted from liver tissues as described (see RT-qPCR protocol up to CDNA reaction). RNA was submitted to Novogene Corporation Inc for library construction and sequencing, with reads aligned to the Mus Musculus reference genome.

### Metabolomics

For the analysis of control, Hlcs^iCre^, Acsf3KO and Acsf3KO/hlcs^iCre^ livers, we submitted five samples of each to Human Metabolome Technologies, Inc, for metabolomic profiling. Compounds were measured using CE-FTMS in both the Cation and Anion modes. The automatic integration software MasterHands was used to process peak data, and software developed at HMT was used for Hierarchical Cluster Analysis (HCA) and Principal Component analysis (PCA). Metabolic pathway maps were developed using VANTED software.

### Acylcarnitine quantification

Acylcarnitines were quantified as described 38, 39, 40. Samples were incubated at 4 °C for 20 min, dried under nitrogen and then 60 μl 3N HCl in n-butanol was added. The samples were incubated for 15 min at 65 °C then dried under LN_2_, and butylated acyl carnitines were reconstituted in 100 μl of mobile phase acetonitrile/water/formic acid (H_2_O:CH_3_CN:HCOOH; 80:19.9:0.1 v/v%). Samples were vortexed, transferred to a centrifuge filter, spun and transferred to an injection vial. Acyl carnitines were analyzed on an API 3200 (AB SCIEX) operated in positive ion mode employing precursor ion scan for m/z 85, which is generated as a characteristic product ion of butyl ester of acyl carnitine species. Quantitation of acyl carnitines was achieved by Chemoview (AB SCIEX) application.

### Proteomics

Flash-frozen liver tissue was submitted to the Johns Hopkins Center for Proteomics Core for total proteome analysis. Samples were prepared in lysis buffer with 8M urea and 50 mM tetraethyl ammoniumbicarbonate. Lysates were treated with LysC and trypsin, then labeled with 10-plex TMT. Peptide fractionation was done using basic pH reverse-phase liquid chromatography, resulting in 24 fractions. Fractions were analyzed using an Orbitrap Fusion Lumos (Thermo Scientific) on an Easy nLC 1200 (Thermo Scientific) using MS1 resolution = 120,000 and MS2 resolution = 30,000, HCD fragmentation method, and MS2 collision energy = 35. Each fraction received a 2-h run time. Total proteome analysis was done in Perseus (version 1.6.0.0) ^60^. *p*-value was calculated by Student’s *t* test. q-value was calculated by significance analysis of microarrays (SAM) and permutation-based false discovery rate (FDR), with SAM S0 value = 0.1.

## Data availability

• RNA-seq data have been deposited at the Gene Expression Omnibus (GEO) with the accession number: GSE305924.
• The mass spectrometry proteomics data have been deposited to the ProteomeXchange Consortium *via* the PRIDE [1] partner repository with the dataset identifier PXD077932.
• *The metabolomics LC-MS data is available at the NIH Common Fund's National Metabolomics Data Repository (NMDR) website, the Metabolomics Workbench,* https://www.metabolomicsworkbench.org *where it has been assigned Project ID PR003100. The data can be accessed directly via it's Project* https://doi.org/10.21228/M8ZW0J. *This work is supported by Metabolomics Workbench/National Metabolomics Data Repository (NMDR) (grant# U2C-DK119886), Common Fund Data Ecosystem (CFDE) (grant# 3OT2OD030544) and Metabolomics Consortium Coordinating Center (M3C) (grant# 1U2C-DK119889).*

## Conflict of interest

The authors declare that they have no conflicts of interest with the contents of this article.
